# Exploring the structural validity and reliability of the swimming competence assessment scale in college students

**DOI:** 10.3389/fpubh.2024.1476732

**Published:** 2024-11-27

**Authors:** Miao Zhao, Cheng Chen, Peng Ning, Bo Wei Zhang, Yupeng Shen

**Affiliations:** ^1^School of Physical Education and Sports Science, South China Normal University, Guangzhou, China; ^2^Department of Sports, Guangzhou Medical University, Guangzhou, China; ^3^Department of Sports, Guangdong University of Foreign Studies, Guangzhou, China

**Keywords:** assessment of swimming competence, structural validity, reliability analysis, college student population, drowning prevention

## Abstract

**Purpose:**

As a globally popular physical activity, swimming also presents challenges due to its inherent aquatic risks. Therefore, the cultivation of swimming competence emerges as a crucial strategy in preventing drowning incidents. This study aimed to develop and validate the Swimming Competence Assessment Scale in College Students (SCAS) to address the gap in structured swimming proficiency evaluation, essential for drowning prevention and water safety education.

**Methods:**

The research involved 160 full-time second-year college students, including 92 males (age: 20.48±0.51 ) and 68 females (age: 20.65±0.70 ), who underwent two swimming ability assessments. The evaluation covered fundamental swimming skills, including entry, submersion, rotation, prone and supine swimming, floating, and exiting the water.

**Results:**

Exploratory Factor Analysis revealed goodness-of-fit for a two-factor model swimming coherent motion and swimming stable posture, which supported the construct validity. The inter-factor construct reliability (CR = 0.866, CR = 0.835) and the square root of Average Variance Extracted ( $\sqrt{AVE}1$ = 0.754, $\sqrt{AVE}1$ = 0.848) exceeded the standards for supporting convergent and discriminant validity. The inter-rater reliability (IRR = 0.542) and Cronbach’s alpha (*α* = 0.840, α = 0.827) coefficient results have demonstrated the internal reliability of the SACS. Positive correlation between SCAS scores at pre-test and post-test provided evidence for SCAS’s test–retest reliability (TRR = 0.825, TRR = 0.758).

**Conclusion:**

SCAS is a valid and reliable assessment scale. It assesses college students’ swimming competence through two aspects: Swimming Coherent Motion and Swimming Stable Posture.

## 1 Introduction

In 2023, the World Health Assembly adopted its inaugural resolution on drowning prevention entitled “Accelerating Global Action on the Prevention of Drowning.” This resolution aimed to mobilize World Health Organization (WHO), governments worldwide, non-governmental organizations, and other partners to address drowning issue—a public health problem that was often overlooked (1). International Life Saving Federation (ILS) released “Position Statement on Swimming and Water Safety Education” which posited that the dissemination of water safety knowledge and fundamental swimming skills could effectively prevent the majority of drowning incidents (2). This statement further accentuated the critical role of swimming skills within water safety education domain. As a globally popular physical activity, swimming also presents challenges due to its inherent aquatic risks. Therefore, the cultivation of swimming competence emerges as a crucial strategy in preventing drowning incidents.

In competitive swimming events, proficiency is determined by measuring the time required to cover a predetermined distance. However, since the issue of drowning cannot be resolved through swift swimming alone, speed and time are insufficient metrics for assessing core competencies in drowning prevention (3). Consequently, researchers have focused on evaluating and determining swimming competence pertinent to aquatic environments. Research has revealed that the development of fundamental aquatic skills and other skills specific to water-based activities is essential for establishing safe relationships with aquatic environments (4). swimming competence encompasses a series of skills designed to foster safer and more harmonious relationships with water environments (5, 6). Researchers from various global locations including New Zealand, Norway, Brazil, and Hong Kong have proposed swimming competencies relevant to aquatic environments, tailored to the specific conditions of water-based activities prevalent in their regions (7–10). Within this body of research, seven fundamental abilities— namely entry into water, submersion, rotation, prone swimming, supine swimming, floating, and exiting the water—are generally regarded as indicators of swimming competence. Concurrently, multiple research works have indicated that the ability to swim continuously in water is only moderately to weakly correlated with these seven swimming competence indicators (11, 12), suggesting that a range of competencies, rather than a single ability to swim forward, might be necessary to effectively prevent drowning.

Swimming competence assessment for drowning prevention is a broad societal and public health concern (13), with in-field technical measurements unavoidably incurring significant time and financial costs. The advantages of scale assessments rely on their ability to be conducted by others as well as for self-evaluation, presenting an effective tool for decreasing the time and economic costs associated with measuring swimming capabilities. Erbaugh (7) was among the pioneers to assess preschool children swimming competence by designing a scale, scoring nine aquatic activities and conducting tests for convergent validity and reliability. However, this scale was deemed overly cumbersome and challenging to implement (11). Hence, researchers have sought to develop more streamlined and assessable scales for swimming competency. Later attempts for the simplification of assessment methods resulted in the development of scales that mostly adopted a binary approach (“can” or “cannot”), failing to precisely measure swimming competence and overlooking many crucial water safety skills (9, 14). Moran et al. (8) performed comprehensive analyses and proposed a series of swimming and water survival skill indicators related to drowning prevention (8). Then, Sundan et al. (15) refined the operability of the assessment based on Delphi method, developing scoring levels for the scale and validating the validity of its contents (15).

Currently, the development and refinement of Swimming Competence Assessment Scales mainly rely on literature review and expert consultation. However, the existing scales still lack sufficient empirical testing procedures for structural validity. When studying and designing Swimming Competence Assessment Scales, ensuring their structural validity is of critical importance. Structural validity presents the degree to which an assessment scale accurately reflects the concept or construct it is intended to measure (16–18). When evaluating swimming competence, it is necessary that the scale be able to comprehensively reflect various aspects of swimming competence, rather than simplifying this complex concept through a singular score. Currently, majority of Swimming Competence Assessment Scales are mainly designed for children, with comparatively fewer approaches available for adults. However, adults are equally at drowning risk (19); therefore, conducting swimming competence assessments within adult populations, particularly in terms of consistency levels, is critical. Research has revealed that humans present significant variability in the strength, speed, agility or skill tests of upper and lower limbs (20). Such variability could affect training load organization (21) and, within the context of swimming competence assessment, could result in individuals overestimating or underestimating their own swimming skills. Consequently, accurate assessment of the consistency of swimming competence aids in precise determination of an individual’s skill level and potential drowning risk, thereby facilitating the provision of more tailored strategies for aquatic safety education and drowning prevention. In aquatic safety education and drowning prevention programs designed for adult swimmers, the accurate assessment of swimming competence consistency becomes particularly critical. This not only enhances the effectiveness of drowning prevention measures but also guarantees that swimmers receive safety guidance suitable for their level and needs, thereby maintaining safety in the water.

University students are also at a high risk for drowning incidents and are potential contributors to societal development. Currently, few research works are available on Swimming Competence Assessment Scales specifically tailored for university students. Investigation on such scales for university students is of utmost importance and offers widespread testing value. Therefore, we hypothesize that the Swimming Competence Assessment Scale (SCAS) for university students possesses strong structural validity, accurately reflecting the multifaceted nature of swimming competence beyond a singular metric. Additionally, we hypothesize that the SCAS demonstrates high reliability, providing consistent results across different testing periods and evaluators. Our research aims to validate these hypotheses, thereby underpinning the formulation of more effective strategies for aquatic safety education and drowning prevention among university students.

## 2 Research subjects and methods

### 2.1 Research subjects

In this research, 160 full-time university sophomores, comprising 92 males and 68 females, were recruited as experimental subjects with their basic demographic information presented in Table 1. Participants were recruited through targeted sampling and had to meet the following inclusion criteria: (a) Being full-time university students. (b) Being 18 years of age or older. (c) Having good health and being without any physical disabilities. (d) Having any level of swimming experience, including none. The subjects underwent two swimming competence assessment tests in a 50-m standard indoor pool (2 m deep), with one-week intervals between tests. Prior to testing, participants were informed of the objectives and specific testing procedures of the experiment, ensuring that all students participated in the experiments with a clear understanding of the process and methodology.

**Table 1:** Basic information of experimental subjects.

Sex	Number/n	Age/Years	Height/CM	Weight/KG
Male	92	20.48±0.51	182.17±6.67	75.43±8.63
Female	68	20.65±0.70	169.65±6.27	59.71±8.38

### 2.2 Research methods

#### 2.2.1 Study design

**Figure 1:**
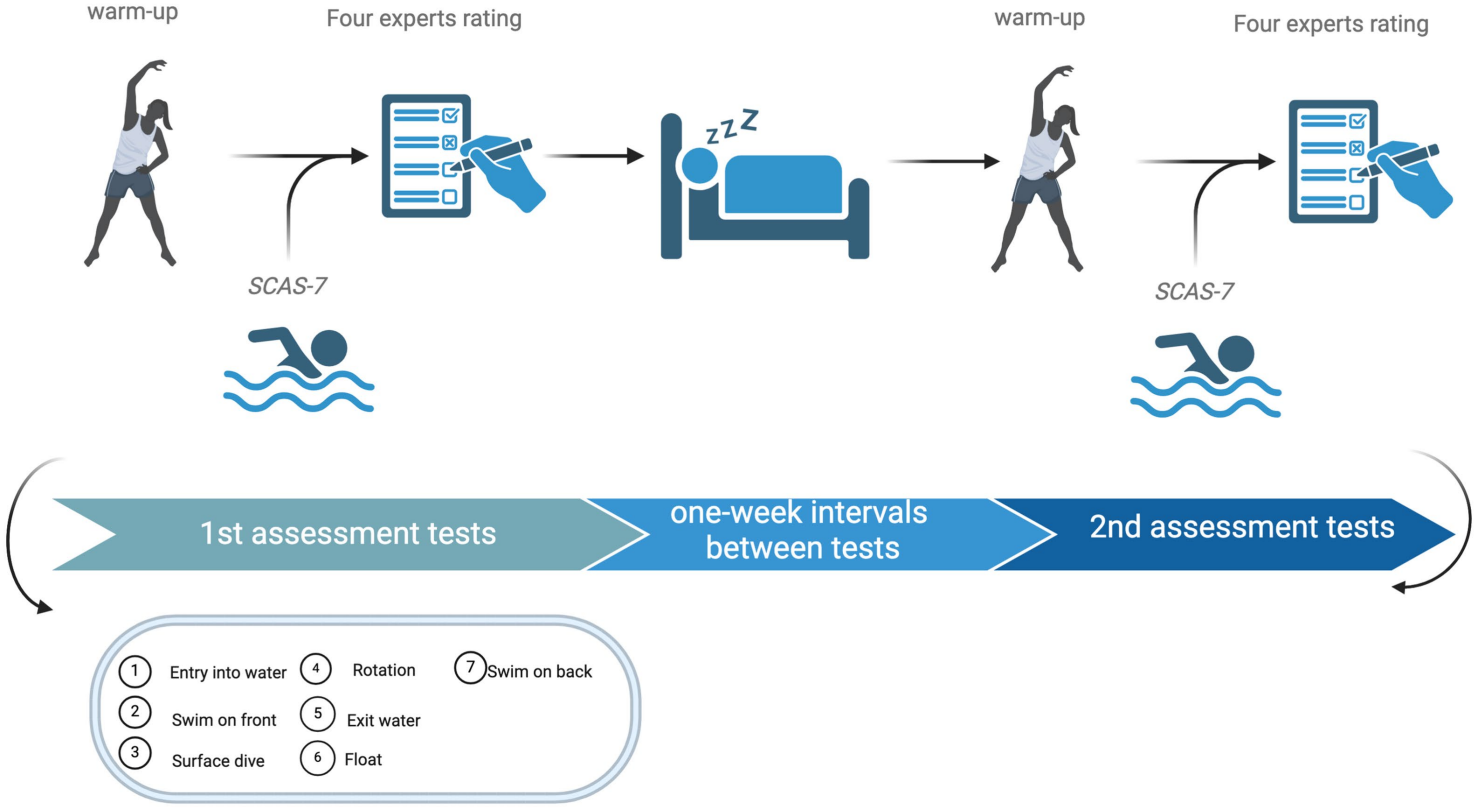
Swimming ability assessment experiment process.

The study employed a cross-sectional design, where participants were briefed on the specific tasks and methods involved in the experiments. During the experimental process, participants warmed up before the tests to prevent injuries. After completing the warm-up, participants performed seven aquatic tasks (Entry into water, Swim on front, Surface dive, Rotation, Exit water, Float, Swim on back) in a 50-meter standard indoor pool with a depth of 2 m. Four experts in the field of swimming evaluated the participants’ performance on these tasks. After a one-week interval without additional swimming practice, the participants underwent a second round of testing in the same environment, evaluated by the same four experts to ensure assessment consistency (as illustrated in Figure 1).

#### 2.2.2 Development of the Swimming Competence Assessment Scale

The research initially developed the Swimming Competency Assessment Scale (SCAS) according to the scale formulated by Sundan et al. (15). Also, insights from a research performed by Stallman et al. (10) were applied to incorporate the ability to perform turns into assessment indicators for the swimming competence of university students. Finally, the following seven swimming competence indicators were identified: water entry, prone progression, floating, submersion, supine progression, rotation, and exiting the water. Depending on the demonstrated proficiency degree, scores were allocated on a scale ranging from 1 to 4 (Table 2).

**Table 2:** Swimming Capacity Assessment Scale (SCAS-7).

Aquatic skill	score	Description
Entry into water	4	Standing entry into deep water, with the body fully submerged and then resurfacing
	3	Squatting at the pool edge or sitting at the pool edge to enter deep water, with the body fully submerged and then resurfacing
	2	Entering deep water from a standing or sitting position at the pool edge, with the body partially or fully submerged, requiring the grasp of the pool edge or a fixed object like a lane divider to resurface
	1	Unable to enter deep water independently, or entering by holding onto a ladder
Swim on front	4	Swim continuously for 100 m or more
	3	Swim continuously for 50–99 m
	2	Swim continuously for 25–49 m
	1	Swim continuously for 0–24 m
Surface dive	4	Complete a dive in one attempt
	3	Attempt to complete a dive in two tries
	2	Attempt to complete a dive three times or more
	1	Unable to complete a dive
Rotation	4	Complete three types of rotations: 1. Rotation around the horizontal axis 2. Rotation around the vertical axis 3. Rotation around the sagittal axis
	3	Complete two out of the three types of rotations: 1. Rotation around the horizontal axis 2. Rotation around the vertical axis 3. Rotation around the sagittal axis
	2	Complete one out of the three types of rotations: 1. Rotation around the horizontal axis 2. Rotation around the vertical axis 3. Rotation around the sagittal axis
	1	Unable to complete body rotation
Exit water	4	Pulling oneself up onto a surface 30 cm high
	3	Complete a surface lift exit in one attempt
	2	Surface lift exit with two or more attempts
	1	Exiting the water using a ladder
Float	4	Float for 5 min
	3	Float for 3 min
	2	Float for 1 min
	1	Float for less than 1 min
Swim on back	4	Swim continuously for 100 m or more
	3	Swim continuously for 50–99 m
	2	Swim continuously for 25–49 m
	1	Swim continuously for 0–24 m

#### 2.2.3 Statistical methods

Data processing and analysis in this research was performed using SPSS software version 26.0, presenting basic information results in the form of mean ± standard deviation (SD). Fleiss-Kappa test was employed to evaluate inter-rater reliability among the four experts. Kappa coefficient (*κ*) was interpreted as follows: κ ≤ 0.40 indicated poor agreement, 0.40 < κ ≤ 0.60 denoted moderate agreement, 0.60 < κ ≤ 0.80 presented substantial agreement, and κ > 0.80 showed almost perfect agreement, at 95% confidence interval (95% CI). Internal consistencies among Swimming Competence Assessment Scale items were evaluated using Cronbach’s alpha (22) coefficient (*α*), with α ≥ 0.7 indicating acceptable consistency level. To assess the stability and reliability of research findings, a test–retest reliability assessment was performed by administering the Swimming Competence Assessment Scale to participants again 1 week after the initial tests were completed. Pearson correlation coefficient (r) was applied for analysis with r values in the range of 0.10–0.29 indicating low correlation, 0.30–0.49 denoting moderate correlation, 0.50–0.69 representing high correlation, 0.70–0.89 showing very high correlation, and 0.90–0.99 revealing nearly perfect linear correlation. This method provided insight into the temporal consistency of the assessment scale.

In addition, exploratory factor analysis was applied to delve into dataset underlying structure. Data suitability for factor analysis was determined using Kaiser-Meyer-Olkin (KMO) measure of sampling adequacy. The KMO statistic value above 0.9 is excellent, between 0.8 and 0.9 indicates good, between 0.7 and 0.8 suggests moderate, between 0.6 and 0.7 denotes fair, between 0.5 and 0.6 implies poor, and below 0.5 is considered unacceptable (23). Factor structure was identified using principal axis factoring with an optimal oblique rotation, based on eigenvalues ≥1 criterion for the determination of factor structure (24). The research used AMOS software version 24.0 software for confirmatory factor analysis (CFA) to validate the factor structure derived from exploratory factor analyses. Traditional fit indices were applied for the evaluation of the model’s fit, including chi-square to degrees of freedom ratio (χ^2^/df), comparative fit index (CFI), Tucker-Lewis index (TLI), root mean square error of approximation (RMSEA), and standardized root mean square residual (SRMR). The critical value for χ^2^/df is considered good if it is greater than 2.00; for the Comparative Fit Index (CFI) and Tucker-Lewis Index (TLI), the critical values are ordinary if greater than 0.90 and good if greater than or equal to 0.95. The critical values for the Root Mean Square Error of Approximation (RMSEA) and the Standardized Root Mean Square Residual (SRMR) are less than 0.08, indicating a good model fit; and an SRMR less than 0.10 suggests that the model is acceptable (25). We evaluated convergent validity using construct reliability (CR) index, where a value exceeding 0.70 denoted good CR. Discriminant validity was assessed through comparing the square roots of the average variance extracted (AVE) with squared correlation coefficients between factors. A scenario where the square roots of AVEs for two factors were greater than the correlation coefficient between them indicated effective discriminant validity between the factors.

## 3 Results

### 3.1 Validity analysis

#### 3.1.1 Structural validity

An exploratory factor analysis was performed on data from 160 swimming competence rating scales. The results indicated a KMO sampling adequacy of 0.761 and a Bartlett’s test sphericity χ^2^-value of 135.871 (*p* < 0.01) suggesting that the obtained data were suitably configured for factor analysis. Using principal axis factoring for factor extraction and considering the assumption of interrelated factors in this research, an oblimin rotation with Kappa set to 4 was applied to extract common factors. Two common factors were identified according to the criterion of eigenvalues greater than or equal to 1 (as presented in Table 3). The cumulative variance explained by these factors was 71.922%, with item communalities ranging from 0.397 to 0.91. The two extracted factors were named “swimming coherent motion factor” and the “swimming stable posture factor.”

**Table 3:** Data table for validity analysis.

Indicator		Factor loading (EFA)	Factor loading (CFA)	CR	AVE	Cr. Alpha	Swimming coherent motion	Swimming stable posture
Swimming coherent motion	Entry into water	0.821	0.710	0.866	0.569	0.840	$\sqrt{AVE}$ _1_ = 0.754	
	Surface dive	0.885	0.862					
	rotation	0.548	0.671					
	Swim on front	0.816	0.881					
	Exit water	0.494	0.610					
Swimming stable posture	Swim on back	0.976	0.929	0.835	0.719	0.827	*R =* 0.594^*^	$\sqrt{AVE}$ _2_ = 0.848
	Float	0.723	0.758					
Total score						0.856	0.955^**^	0.757^**^

^*^p < 0.05, ^**^P < 0.01. ^*^Entry into Water: Assess the ability to enter the water with a complete submersion, followed by a controlled resurfacing and proper orientation; Surface Dive: Assess the ability to dive from the surface to the pool floor, retrieve an object (such as a diving ring), and resurface, testing depth and pressure adaptation; Rotation: Test the subject’s rotation ability in 3 directions (horizontal axis, vertical axis, sagittal axis); Swim on Front: Evaluate the capability to level off the body and achieve continuous progression through effective propulsion while swimming on the front; Exit Water: Test the ability to exit deep water and simulate climbing onto a dock or other elevated surfaces without the use of feet, ensuring safety and self-assistance in exiting the pool; Swim on Back: Evaluate the skill to level off and maintain continuous progression through propulsion while swimming on the back; Float: Measure the skill of floating to rest and conserve energy, optimizing buoyancy and relaxation.

**Figure 2:**
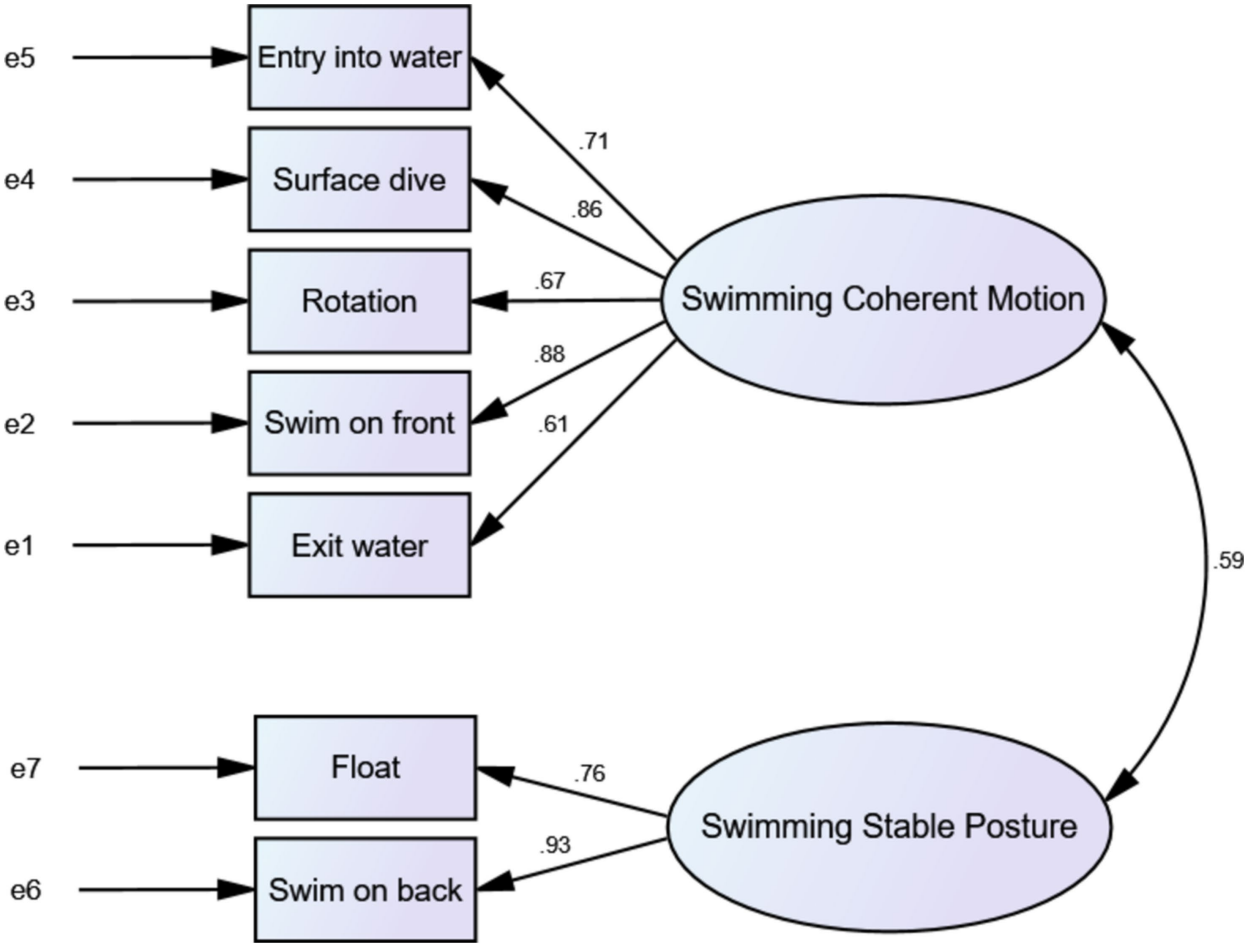
Confirmatory factor analysis data plot.

Confirmatory factor analysis was applied in this research to evaluate the fit of a two-factor model for swimming competence test. The obtained results were as follows: χ^2^/df (17.004/13) = 1.308, RMSEA = 0.089, SRMR = 0.0627, NFI = 0.885, CFI = 0.968, and GFI = 0.902. All metrics either met or approached the fit standards established in psychometrics, indicating that the goodness of fit of the developed model complied with statistical standards. By comprehensive consideration, it was concluded that the overall fit of the measurement model was consistent with theoretical expectations, also suggesting that the scale demonstrated good structural validity (as illustrated in Figure 2). A two-dimensional Swimming Competence Assessment Scale was reformulated based on construct validity (as presented in Table 4).

**Table 4:** Swimming Competence Assessment Scale.

Swimming competence	Swimming coherent motion																				Swimming stable posture							
Activities	Entry into water				Surface dive				Rotation				Swim on front				Exit water				Float				Swim on back			
Score	4	3	2	1	4	3	2	1	4	3	2	1	4	3	2	1	4	3	2	1	4	3	2	1	4	3	2	1
																												

#### 3.1.2 Convergent and discriminant validity

Concerning convergent validity, CR for each factor of swimming competence test ranged from 0.835 to 0.866, all surpassing the threshold of 0.70, indicating satisfactory CR of the measurement model. Regarding discriminant validity, the study findings revealed that Factor 1 had an AVE of 0.569 and a CR of 0.754, while corresponding values for Factor 2 0.719 and 0.848, respectively. The correlation coefficient between the two factors was 0.594. Since AVE square roots for both factors were higher than the correlation coefficient between the constructs, it signified that the latent variables were distinct, confirming good discriminant validity between the two factors (as given in Table 3).

### 3.2 Reliability analysis

#### 3.2.1 Rater consistency

Fleiss-Kappa consistency tests were performed on the swimming competence scores of 160 students, as rated by four expert judges. The overall Fleiss-Kappa value was found to be 0.542 (*p* < 0.01), indicating moderate consistency. Hence, it could be inferred that there was a certain agreement level among the four expert raters regarding the assessment of students’ swimming competence. A consistency analysis was conducted on different swimming competence indicators of the 160 students. Fleiss-Kappa values for entry into water and rotational ability, rated by the four evaluators, were 0.628 and 0.616 (*p* < 0.01), respectively, indicating high consistency. For prone ability, floating ability, submersion ability, supine ability, and exiting water ability, Fleiss-Kappa values among the four evaluators were all greater than 0.8 (*p* < 0.01), indicating a high degree of consistency (as presented in Table 5).

**Table 5:** Fleiss kappa test consistency analysis of the four evaluators’ swimming competence rating scale.

	Kappa	SD	z	*p*-value	95% CI
Entry into water*	0.628	0.046	13.688	<0.0001	0.625–0.631
Swim on front*	0.982	0.040	24.791	<0.0001	0.980–0.985
Float/rest*	0.855	0.045	19.160	<0.0001	0.852–0.858
Surface dive*	0.951	0.055	17.283	<0.0001	0.947–0.954
Swim on back*	0.918	0.043	21.425	<0.0001	0.915–0.920
rotation*	0.616	0.040	15.310	<0.0001	0.614–0.619
Exit water*	0.894	0.062	14.363	<0.0001	0.890–0.898
Totals*	0.542	0.017	32.827	<0.0001	0.541–0.543

^*^Entry into Water: Assess the ability to enter the water with a complete submersion, followed by a controlled resurfacing and proper orientation; Surface Dive: Assess the ability to dive from the surface to the pool floor, retrieve an object (such as a diving ring), and resurface, testing depth and pressure adaptation; Rotation: Test the subject’s rotation ability in 3 directions (horizontal axis, vertical axis, sagittal axis); Swim on Front: Evaluate the capability to level off the body and achieve continuous progression through effective propulsion while swimming on the front; Exit Water: Test the ability to exit deep water and simulate climbing onto a dock or other elevated surfaces without the use of feet, ensuring safety and self-assistance in exiting the pool; Swim on Back: Evaluate the skill to level off and maintain continuous progression through propulsion while swimming on the back; Float: Measure the skill of floating to rest and conserve energy, optimizing buoyancy and relaxation.

#### 3.2.2 Internal consistency reliability

For factors representing two distinct concepts, the reliability of subscales was calculated. Reliability coefficient was 0.840 for swimming coherent motion and was 0.827 for swimming stable posture (with Cronbach’s alpha coefficients >0.70). This indicated a good internal consistency within the scale. Table 3 presents detailed results.

#### 3.2.3 Test–retest reliability

Test–retest reliability was explored by having 160 subjects retake swimming competence tests 1 week later. The obtained coefficients for the seven indicators were as follows: entry into water 0.393*, prone progression 0.802**, floating 0.667**, submersion ability 0.760**, supine progression 0.636**, rotation 0.654**, and exiting water 0.568**; with test–retest reliability for swimming coherent motion at 0.824** and for swimming stable posture at 0.758**. Test–retest reliability coefficients for these seven indicators revealed moderate to high correlation and reliability coefficients for both factors were greater than 0.700, indicating that the scale exhibited acceptable stability.

## 4 Discussion

Validity and reliability tests were conducted in this research on the structure and reliability of the developed swimming assessment scale through model fitting tests. It was found that the indicators of swimming assessment scale could be categorized into two main aspects: swimming coherent motion ability and swimming stable posture ability. Both aspects exhibited good convergent and discriminant validity. The swimming assessment scale demonstrated good reliability in both rater consistency and internal consistency tests. However, in test–retest reliability assessments, apart from prone progression and submersion abilities, which showed good test–retest reliability, remaining indicators (entry into water, floating, supine, rotation, and exiting water) did not exhibit good test–retest reliability.

Structural validity analysis is a pivotal element in assessing the quality of evaluative tools, as it relates to the alignment between assessment outcomes and actual swimming competence (18). Using exploratory and confirmatory factor analyses, the scale was found to encompass two common factors, indicating that assessment of swimming competence relevant to water safety could be divided into two main components. In the structural validity examination, the first component encompassed five actions related to post-immersion movement abilities; i.e., entry into water, diving, rotation, prone progression, and exiting water. These five actions were essential phases in the behavior of falling into water. The second part included two indicators: supine progression and floating, which were essential skills for staying longer in water after falling in, waiting for rescue, and stabilizing one’s position. Convergent and discriminant validity test results further validated that these two parts possessed good structural validity. In previous studies, researchers have often categorized swimming competence into six or more dimensions for assessment and measurement purposes (11, 15). However, the two-part structure suggested in this research more accurately reflected the effective structure of the swimming competence of college students. Identification of structural validity not only enhanced the scientific nature of the scale, but also robustly supported its practical applicability. The actions of entry into water, submersion, rotation, prone progression, and exiting water were characterized by a dynamic sequence of movements, which could be considered as a complex series of motions constituting a sportive task (26). Previous research works have repeatedly emphasized that the evaluation of swimming competence should focus on dynamism and coherence, rather than on isolated movements (10, 12, 15). Therefore, structural validation discriminated between the competencies of dynamic coherent motion and static stability in posture within aquatic ability assessment. Concurrently, antecedent research delineates swimming distance and swimming skill as the two principal categories of aquatic proficiency (11). However, our research revealed that while prone and supine swimming both reflected capabilities in advancing through water, thus indicating swimming distance capacity, they were categorized into two distinct structures within swimming competence related to aquatic safety. This divergence stemmed from significant differences in buoyancy and the rotational effects of trunk kinematics between supine and prone swimming (27). Specifically, supine abilities offered an advantage in prolonging water retention, whereas prone capabilities facilitated more effective progression through water (28). Consequently, prone abilities were categorized within a unified characteristic of swimming coherent motion abilities, aligning with entry into water, submergence, rotation, and emerging onto land, whereas supine swimming capacity was akin to the capability of prolonged water retention, consistent with the competencies of swimming stable posture abilities.

Scale reliability analysis was a critical component in the evaluation of the quality of assessment tools. The obtained results pertaining to inter-rater consistency within this research indicated that the reliability of the Swimming Competence Assessment Scale across different evaluators was acceptable. This suggested that as long as evaluators adhered to the same procedures and standards, assessment outcomes were fundamentally consistent regardless of the assessor. Such consistency ensured the stability and reliability of assessment results, thereby enhancing scale credibility. Internal consistency results within the study reflected the homogeneity of the developed Swimming Competence Assessment Scale in measuring the swimming capabilities of university students, with all indicators effectively reflecting the aquatic competencies of these individuals. Despite the high reliability of the developed scale, the test–retest reliability of the five indicators—entry into water, floating, supine swimming, rotation, and emerging onto land—demonstrated low consistency between the two tests. This discrepancy might be attributed to the dynamic nature of swimming competence, which evolved with the acquisition of skills and development of physical capacities. The unique characteristics of the aquatic environment endow swimming competence with environmental adaptability (17, 29). The accumulation of time spent in aquatic activities distinctly influenced the enhancement of swimming capabilities. The accumulation of time spent engaging in activities within aquatic environments exhibited significant variance in its impact on the enhancement of swimming capabilities (21, 30). Water entry and exit indicators represent the ability to enter and leave an aquatic environment, while floating, supine ability, and rotational ability signified the capacity for body posture control within water (26). These indicators were intimately associated with adaptation to aquatic environments (29). On the other hand, the test–retest reliability of prone swimming and diving capabilities, which demanded more intricate motor techniques (26), appeared to be more stable. Proficiency in these motor skills necessitated prolonged periods of learning or practice for improvement. This resulted in their greater stability across two capability assessments.

Through the examination of swimming competence scale, we discovered that this scale was equally applicable to college student population, capable of objectively reflecting the actual swimming competence of university students. However, in the context of drowning prevention and swimming instruction among college students, further consideration should be given to two groups of characteristics. Emphasis should be placed on coherence and tight correlation of water entry, submersion, rotation, prone swimming, and exiting the water in both drowning prevention and swimming teaching. Simultaneously, the necessity of enhancing backstroke and floating abilities in teaching processes should be heightened to effectively augment students’ capabilities in water while awaiting rescue. The findings of this research reminded us that swimming competence assessment should not be regarded as a long-term fixed state but as a dynamic process. Therefore, the developed scale was more suited for assessing swimming capabilities at specific points in time rather than representing long-term swimming competence. In addition, considering the dynamic nature of swimming competence, the proposed swimming competence scale can be periodically applied during the teaching process for real-time feedback on swimming capabilities, which makes it a valuable diagnostic tool in the process of learning swimming skills.

### 4.1 Limitations and future directions

#### 4.1.1 Research limitations

The test design in this study primarily assessed students’ immediate responses to aquatic skills, failing to comprehensively capture the long-term development of swimming competence or changes across different training stages. Therefore, future research should employ multiple assessments and longitudinal tracking to validate the long-term effectiveness of the SCAS.

Although the SCAS covers basic swimming skills performed in a pool, it does not include assessments of broader water safety competencies, such as emergency responses in natural water bodies or under specific conditions (e.g., swimming in clothing). This limitation may reduce the SCAS’s applicability in evaluating comprehensive swimming competence. Future studies should consider incorporating a wider range of assessment criteria to enhance the scale’s practical value.

#### 4.1.2 Practical applications

The SCAS can serve as a standardized assessment tool in college swimming courses. By regularly evaluating students’ swimming competence, instructors can adjust the course content based on student performance, ensuring that students meet the expected skill levels by the end of the course.

Colleges can integrate the SCAS into water safety education programs to assess and enhance students’ water safety skills. Systematic evaluation of swimming competence will enable schools to conduct more targeted drowning prevention education, improving students’ safety awareness and emergency response capabilities.

The SCAS can be incorporated into college students’ physical fitness assessment systems as part of evaluating their overall physical abilities. This will help schools gain a more comprehensive understanding of students’ physical fitness and provide a basis for developing more personalized exercise programs.

## 5 Conclusion

College students’ Swimming Competence Assessment Scale can be utilized for self-assessment or by others to explore the swimming competence of college students, demonstrating consistency across different evaluators. The swimming capabilities of college students were divided into two aspects: coherent swimming actions and stable swimming postures. In daily teaching and training, as well as drowning prevention education, it is necessary to address and evaluate college students’ swimming competence from these two dimensions in order to further enhance the cultivation of drowning prevention skills among college students. The developed college students’ swimming competence scale exhibited variability in repeated measurements, indicating that swimming competence evolved with the accumulation of time spent in aquatic activities. The assessment of college students’ swimming competence should dynamically be adjusted as swimming skills improved and adaptability to aquatic environments increased. The developed college students’ swimming competence scale could be extensively applied in the actual teaching and assessment of swimming competence, further advancing the development of education related to swimming and aquatic capabilities.

## Data Availability

The original contributions presented in the study are included in the article/supplementary material, further inquiries can be directed to the corresponding author.

## References

[1] International Life Saving Federation. (2023). WHA resolution - accelerated action on global drowning prevention. Available at: https://wcdp2023.com/2023/05/30/elementor-1446/ (Accessed March 22).

[2] International Life Saving Federation. (2008). Clear link between drowning prevention and education. Available at: https://www.ilsf.org/2008/01/07/clear-link-between-drowning-prevention-and-education/ (Accessed March 22).

[3] Zhang H Wang B Luo S Xia W. Exploring factors influencing students water high-risk practices based on grounded theory. China Safety Sci J. (2017) 27:7–12. doi: 10.16265/j.cnki.issn1003-3033.2017.03.002

[4] Canossa S Fernandes RJ Carmo C Andrade A Soares SM. Ensino multidisciplinar em natação: reflexão metodológica e proposta de lista de verificação. Motricidade. (2007) 3:82–99. doi: 10.6063/motricidade.3(4).656

[5] Langendorfer SJ. Considering drowning, drowning prevention, and learning to swim. Int J Aquat Res Educ. (2011) 5:2. doi: 10.25035/ijare.05.03.02

[6] Quan L Ramos W Harvey C Kublick L Langendorfer S Lees TA. Toward defining water competency: an American red cross definition. Int J Aquat Res Educ. (2015) 9:3. doi: 10.25035/ijare.09.01.03

[7] Erbaugh SJ. (1978). Assessment of swimming performance of preschool children. Perceptual and Motor Skills, 47, 1179–1182. doi: 10.2466/pms.1978.46.3f.1179 745893

[8] Moran K Stallman RK Kjendlie P-L Dahl D Blitvich JD Petrass LA. Can you swim? An exploration of measuring real and perceived water competency. Int J Aquat Res Educ. (2012) 6:4. doi: 10.25035/ijare.06.02.04

[9] Murcia JAM. Desarrollo y validación preliminar de escalas para la evaluación de la competencia motriz acuática en escolares de 4 a 11 años. (Development and preliminary validation of an aquatic competence scale for children 4 to 11 years old). RICYDE. (2005) 1:14–27. doi: 10.5232/ricyde2005.00102

[10] Stallman RK Moran Dr K Quan L Langendorfer S. From swimming skill to water competence: towards a more inclusive drowning prevention future. Int J Aquat Res Educ. (2017) 10:3. doi: 10.25035/ijare.10.02.03

[11] Chan DKC Lee ASY Macfarlane DJ Hagger MS Hamilton K. Validation of the swimming competence questionnaire for children. J Sports Sci. (2020) 38:1666–73. doi: 10.1080/02640414.2020.1754724 32321367

[12] Stallman B. W. L. A. (2016). The validity of swimming speed as a predictor of swimming competence.

[13] Meddings DR Scarr J-P Larson K Vaughan J Krug EG. Drowning prevention: turning the tide on a leading killer. Lancet Public Health. (2021) 6:e692–5. doi: 10.1016/S2468-2667(21)00165-1, PMID: 34310906 PMC8391011

[14] Junge M. Blixt T. Stallman R. (2010). The construct validity of a traditional 25m test of swimming competence. In. Kjendlie PL Reder RK, Biomechanics and Medicine in Swimming XI (S. 331-332). Oslo: Norwegian School of Sport Sciences.

[15] Sundan J Haga M Lorås H. Development and content validation of the Swimming Competence Assessment Scale (SCAS): a modified Delphi study. Percept Mot Skills. (2023) 130:1762–80. doi: 10.1177/00315125231177403, PMID: 37202396 PMC10363955

[16] Li B Liu Y. Research on the development and verification of Children's fundamental motor skills test system based on physical literacy evaluation. China Sport Sci. (2022) 42:31–42. doi: 10.16469/j.css.202204004

[17] Liu T Wassell N Liu J Zhang M. Mapping research trends of adapted sport from 2001 to 2020: a bibliometric analysis. Int J Environ Res Public Health. (2022) 19:12644. doi: 10.3390/ijerph191912644, PMID: 36231944 PMC9564994

[18] Zhang L. (2000). Research methods of sports science. Beijing: Higher Education Press.

[19] Zhang H Yun LZ Luo S. Effects of hazards and sensation-seeking on intermediate swimming college students’ hazard perceptions. Brain Behav. (2023) 13:e3338. doi: 10.1002/brb3.3338, PMID: 38031238 PMC10726776

[20] Reichmuth D Olstad BH Born D-P. Key performance indicators related to strength, endurance, flexibility, anthropometrics, and swimming performance for competitive aquatic lifesaving. Int J Environ Res Public Health. (2021) 18:3454. doi: 10.3390/ijerph18073454, PMID: 33810445 PMC8038010

[21] Born D-P Lorentzen J Björklund G Stöggl T Romann M. Variation vs. specialization: the dose-time-effect of technical and physiological variety in the development of elite swimmers. BMC Res Notes. (2024) 17:48. doi: 10.1186/s13104-024-06706-x, PMID: 38355679 PMC10865614

[22] Cronbach LJ. Coefficient alpha and the internal structure of tests. Psychometrika. (1951) 16:297–334. doi: 10.1007/BF02310555

[23] Wu M. (2013). Structural Equation Models: Operation and Application of AMOS. Chongqing: Chongqing University Press.

[24] Hair JF Black WC Babin BJ Anderson RE. (2018). Multivariate Data Analysis. 8th edition. Andover, Hampshire: Cengage Learning EMEA.

[25] Hu L Bentler PM. Cutoff criteria for fit indexes in covariance structure analysis: conventional criteria versus new alternatives. Struct Equ Model Multidiscip J. (1999) 6:1–55. doi: 10.1080/10705519909540118

[26] Magill RA. (2006). Motor skill learning and control (Z. Zhang, Trans.). Beijing: China Light Industry Press.

[27] Gonjo T Fernandes RJ Vilas-Boas JP Sanders R. Differences in the rotational effect of buoyancy and trunk kinematics between front crawl and backstroke swimming. Sports Biomech. (2023) 22:1590–601. doi: 10.1080/14763141.2021.1921835, PMID: 34009106

[28] Costa MJ Barbosa TM Morais JE Miranda S Marinho DA. Can concurrent teaching promote equal biomechanical adaptations at front crawl and backstroke swimming? Acta Bioeng Biomech. (2017) 19:81–8. doi: 10.5277/ABB-00511-2015-03, PMID: 28552928

[29] Seifert L Komar J Barbosa T Toussaint H Millet G Davids K. Coordination pattern variability provides functional adaptations to constraints in swimming performance. Sports Med. (2014) 44:1333–45. doi: 10.1007/s40279-014-0210-x 24895244

[30] Zhao M. Du K. Shen Y. (2021). The influence of different length of adaptive skills learning in college students' breaststroke learning based on the Macmillan analysis approach.

